# A Web-Based Public Health Intervention for Addressing Vaccine Misinformation: Protocol for Analyzing Learner Engagement and Impacts on the Hesitancy to Vaccinate

**DOI:** 10.2196/38034

**Published:** 2022-05-30

**Authors:** Leigh Powell, Radwa Nour, Youness Zidoun, Sreelekshmi Kaladhara, Hanan Al Suwaidi, Nabil Zary

**Affiliations:** 1 Institute for Excellence in Health Professions Education Mohammed Bin Rashid University of Medicine and Health Sciences Dubai United Arab Emirates; 2 College of Medicine Mohammed Bin Rashid University of Medicine and Health Sciences Dubai United Arab Emirates

**Keywords:** public health, population health, education, gamification, COVID-19, vaccination, misinformation, infodemic, vaccine hesitancy, web-based health, web-based intervention, learning design, dissemination

## Abstract

**Background:**

A barrier to successful COVID-19 vaccine campaigns is the ongoing misinformation pandemic, or infodemic, which is contributing to vaccine hesitancy. Web-based population health interventions have been shown to impact health behaviors positively. For web-based interventions to be successful, they must use effective learning design strategies that seek to address known issues with learner engagement and retention. To know if an intervention successfully addresses vaccine hesitancy, there must be some embedded measure for comparing learners preintervention and postintervention.

**Objective:**

This protocol aims to describe a study on the effectiveness of a web-based population health intervention that is designed to address vaccine misinformation and hesitancy. The study will examine learner analytics to understand what aspects of the learning design for the intervention were effective and implement a validated instrument—the Adult Vaccine Hesitancy Scale—to measure if any changes in vaccine hesitancy were observed preintervention and postintervention.

**Methods:**

We developed a fully web-based population health intervention to help learners identify misinformation concerning COVID-19 and share the science behind vaccinations. Intervention development involves using a design-based research approach to output more effective interventions in which data can be analyzed to improve future health interventions. The study will use a quasi-experimental design in which a pre-post survey will be provided and compared statistically. Learning analytics will also be generated based on the engagement and retention data collected through the intervention to understand what aspects of our learning design are effective.

**Results:**

The web-based intervention was released to the public in September 2021, and data collection is ongoing. No external marketing or advertising has been done to market the course, making our current population of 486 participants our pilot study population. An analysis of this initial population will enable the revision of the intervention, which will then be marketed to a broader audience. Study outcomes are expected to be published by August 2022. We anticipate the release of the revised intervention by May 2022.

**Conclusions:**

Disseminating accurate information to the public during pandemic situations is vital to contributing to positive health outcomes, such as those among people getting vaccinated. Web-based interventions are valuable, as they can reach people anytime and anywhere. However, web-based interventions must use sound learning design to help incentivize engagement and motivate learners to learn and must provide a means of evaluating the intervention to determine its impact. Our study will examine both the learning design and the effectiveness of the intervention by using the analytics collected within the intervention and a statistical analysis of a validated instrument to determine if learners had a change in vaccine hesitancy as a result of what they learned.

**International Registered Report Identifier (IRRID):**

DERR1-10.2196/38034

## Introduction

### Background

In 2020, the world began to face the challenges of COVID-19. As the year shifted to 2021, a new hope emerged in the form of a vaccine, and with that came concerns from the public over the role that vaccines play in public health and the safety of vaccines, leading to the emergence of an infodemic of misinformation on the internet and social media [1]. Recent studies have suggested that social media platforms act as a medium for health misinformation to spread more easily than scientific knowledge [2,3]. The definition of *health misinformation* is “a health-related claim that is based on anecdotal evidence, false, or misleading owing to the lack of existing scientific knowledge” [4,5]. *Health misinformation* is a term that considers false information that does not aim at harming others but has had an apparent role in the recent pandemic. These concerns and others have contributed to a phenomenon known as *vaccine hesitancy*, which is defined as a “delay in acceptance or refusal of vaccination despite the availability of vaccination services” [6]. Health communication has been shown to play an essential role in combating behaviors related to vaccine hesitancy [6,7]. When health communication is poor, it can undermine individuals’ confidence in a vaccine, leading to vaccine hesitancy. Effective health communication strategies are proactive and ensure the accuracy and reliability of information by using means that are easily accessible to the public. Factors that influence the success of health communication strategies include providing mechanisms through which individuals can receive information, communicate their needs, connect with others, and mobilize community engagement [6]. One way of using health communication is through public health education interventions. Public health education interventions successfully raise awareness about public health concerns and change behaviors and perspectives toward various diseases [8]. For example, education interventions have played a vital role in the prevention and control of communicable diseases, such as SARS (severe acute respiratory syndrome) [9] and MERS (Middle East respiratory syndrome) [10], by aiding in improving learner anxiety, depression, and fear [9]. It stands to reason that public health education may also positively impact vaccine hesitancy related to COVID-19 vaccines.

Health education interventions aim to improve the access to and delivery of information to address social determinants of health and empower behavior change [11]. There are many different approaches to developing interventions, including individual methods, group methods, and mass media methods [12]. Each of these methods has its own benefits that aid its effectiveness and its own barriers [13]. Web-based health education interventions are useful in reaching a broad audience, as they can overcome physical barriers, enabling education to be accessible anywhere and anytime. However, web-based interventions come with their challenges. As demonstrated by research in massive open online courses (MOOCs), completion rates for web-based interventions vary greatly, and such interventions tend to have high attrition rates [14]. A lack of learner motivation and a lack of interactivity have been reported as contributing factors [15]. Paying attention to the design of learning is a way of engaging and motivating learners to achieve learning objectives [14]. Learning design is the process of designing education by giving thoughtful consideration to content and activities for describing, understanding, supporting, and guiding the practices and processes of learning [16]. Given that enrollment in web-based interventions is only expected to rise, learning designs must implement effective strategies to promote learner engagement, motivation, and learning outcomes.

The Institute for Excellence in Health Professions Education (ieHPE) at the Mohammed Bin Rashid University of Medicine and Health Sciences (MBRU) has a history of implementing effective and successful web-based population health education interventions. For example, our *Community Immunity Ambassador* series, which was designed to engage the public in understanding, preventing, and coping with the COVID-19 pandemic, went viral, with over 1 million learners engaging with these initiatives. Over time, the ieHPE has systematized its way of designing and developing initiatives to incorporate strategies for educating learners and motivating and empowering them to share information with others. The ieHPE uses an iterative approach to design and develop interventions that involves using small, multidisciplinary teams consisting of educational experts, subject matter experts (SMEs), and digital content creators.

### Objective

The objective of this protocol is to describe the development of a web-based educational intervention that is designed to address vaccine misinformation and the proposed analysis of data to understand if there was any impact on learners’ hesitancy to vaccinate. The intervention will use a pre-post test design involving the use of a validated instrument called the *Adult Vaccine Hesitancy Scale* (aVHS) [17] to measure changes in vaccine hesitancy. Each learner will complete the aVHS at the beginning and end of the intervention. In addition, the analytics collected about how users interacted with the intervention will also be analyzed to understand how to make the intervention more effective.

Our research questions include the following:

1. What kinds of learner engagement did we observe in the course (ie, use patterns, levels of engagement, and completion rates)?
2. What changes are observed in a participant’s vaccine hesitancy status as a result of the learning, as determined by a pre-post validated survey tool (aVHS)?
3. What kind of improvements or alterations are recommended to the course based on our observations?

## Methods

The methods for our study are described below in 2 parts. The first part describes the methodology behind the design and development of the web-based intervention, and the second part describes the aVHS instrument [17] and analysis of the data that we will collect.

### Design and Development of the Web-Based Intervention

Our web-based intervention is publicly available at no cost on our web-based course platform—MBRU Learn. The intervention was developed to educate learners about (1) how to mitigate becoming infected with SARS-CoV-2, (2) how to spot and prevent the sharing of misinformation and disinformation related to COVID-19 and vaccines, and (3) the science behind vaccinations and their impact on public health. The intervention uses learning design principles to motivate learners to complete the intervention and empower them to share their new knowledge and achievements. The Center for Learning and Teaching methodology for intervention design includes keeping initial project teams small and agile to allow for quicker turnaround times. Several prototypes were created iteratively among this small team, which were sent to a larger pool of SMEs and testers for review and feedback. The three core members of this project team consisted of an SME, an educational expert, and a digital content developer. In addition, our SME was a physician with expertise in public health and vaccination outreach.

**Figure 1:**
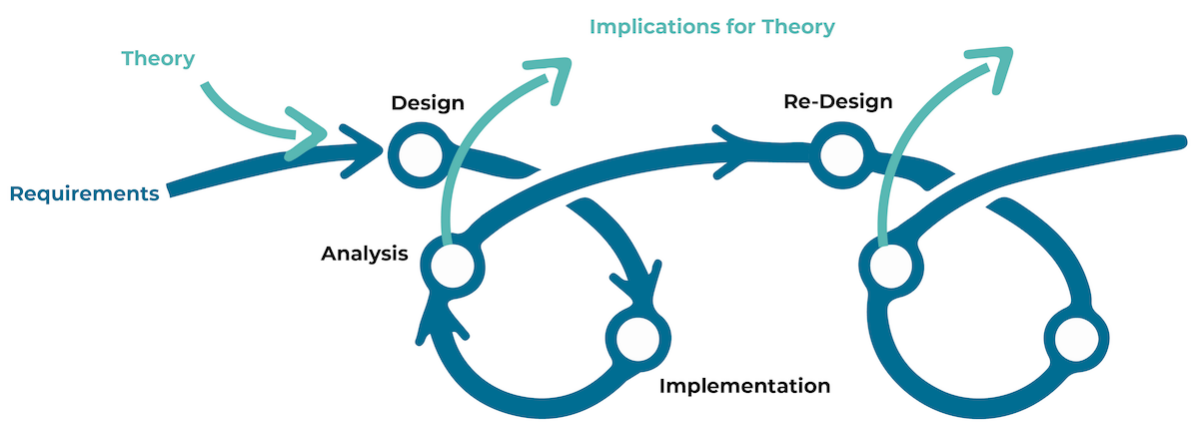
Design-based research process, as adapted from Fraefel [20].

The Center for Learning and Teaching strategy draws on principles from design-based research (DBR), in which successive iterations of design, analysis, implementation, and reflection are used to ensure the practical application and success of the intervention (Figure 1) [18,19]. DBR has been described as a practical approach to “bringing about transformation through the design and use of solutions to real problems” [20]. The guiding principles of DBR include (1) inquiry into a problem of practice to generate usable knowledge to inform intervention and theory, (2) close collaboration between researchers and practitioners in the design and development of interventions (ie, new tools, instructional materials, etc), and (3) the use of iterative design cycles for the design and continuous improvement of interventions [18,20,21]. Using DBR rather than an instructional design model, such as the Analyze, Design, Develop, Implement, and Evaluate approach [22] or the successive approximations model [23], helps to better ensure that development and research stay closely linked, creating an opportunity to contribute to both theory and practice.

### Description of Phases

The requirements phase consisted of a project meeting for discussing the intervention. Our requirements phase determined the learning objectives, audience, and platform for the intervention, as well as the time requirements for the intervention’s release and the data we will analyze to determine if we are successful. The learning objectives are detailed as follows: at the end of the course, the learner should be able to (1) describe the negative impact that misinformation has on the pandemic, (2) evaluate information to avoid misinformation traps, (3) apply strategies to reduce the risk of contracting and spreading COVID-19, and (4) recognize the science behind vaccinations and how they work to protect public health.

The platform for dissemination will be the MBRU’s public-facing, web-based platform, which is called *MBRU Learn*. The platform offers rapid course development services, simple registration and marketing methods, and the ability to collect various types of learner engagement and assessment information.

**Figure 2:**
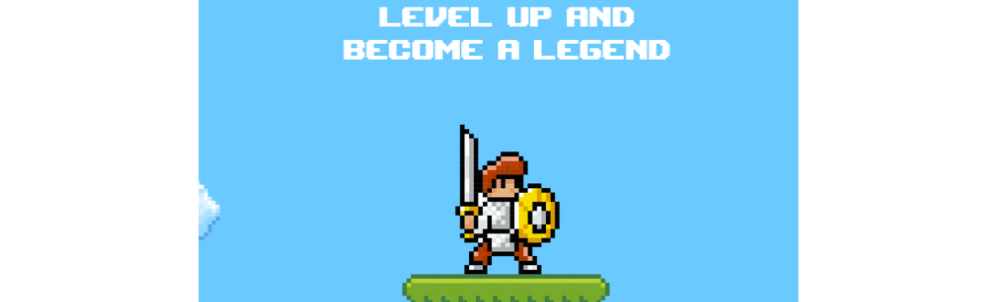
Screenshot from the educational intervention—participants' avatar in its final state.

**Figure 3:**
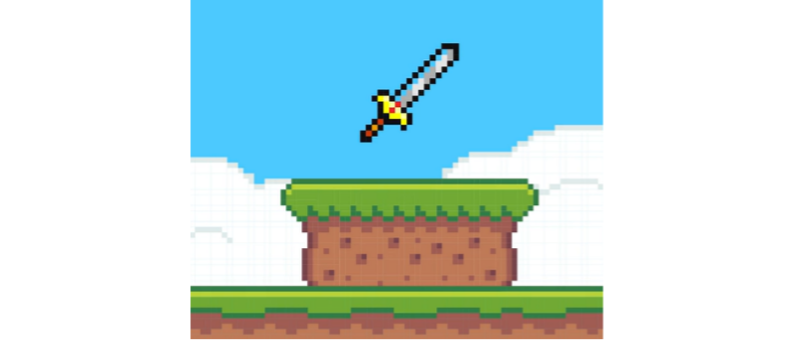
Screenshot from the educational intervention—collection of objects.

**Figure 4:**
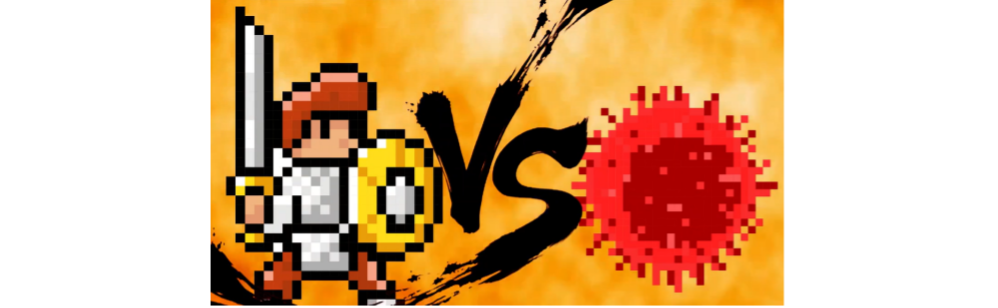
Screenshot from the educational intervention—final "big boss" challenge.

After the requirements phase, our team entered the design phase, in which we ideated on a theme for the course and the learning design strategies. We determined that we would use gamification strategies in both the learning design and the graphic development process. The gamification techniques in the intervention include using graphics and media to simulate a fun, video game–like experience (Figure 2) and using elements such as progress tracking through the collection of objects (Figure 3) and demonstrating growth by moving up in levels toward the final level, in which the learner slays the “big boss” (Figure 4). The intervention concludes with the awarding of a certificate and an invitation to a social media campaign, in which the learner can share achievements with others by using a unified hashtag.

**Figure 5:**
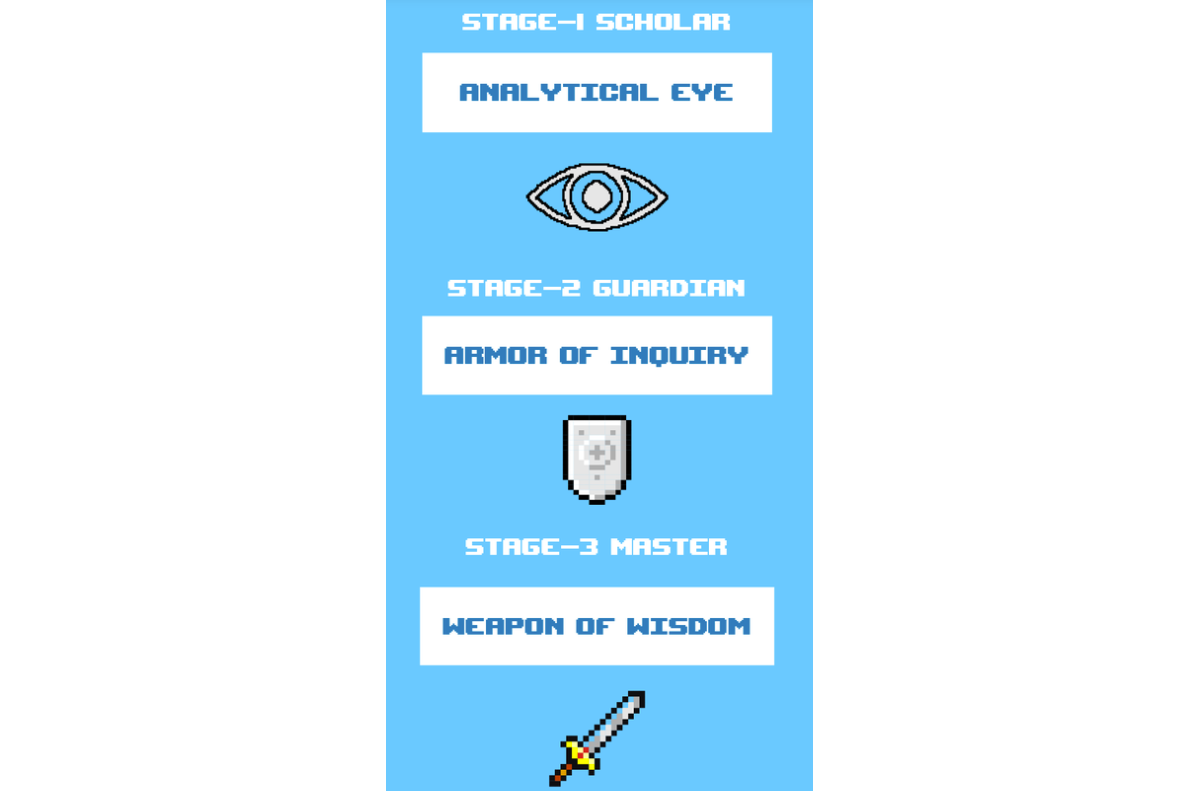
Screenshot from the educational intervention—ranks and equipment for each stage.

We iterated through several storyboards and prototypes during the design phase, and each iteration grew in in terms of the complexity of the content and graphics until a fully developed beta intervention was achieved and sent to additional SMEs for feedback. Feedback from the SMEs was then incorporated into the next iteration to reach the version that was implemented on MBRU Learn. The final, implemented version of the intervention consists of the following three chapters: (1) *Understanding and counteracting misinformation*, (2) *COVID-19 transmission and prevention*, and (3) *The science of vaccinations*. A combination of media is used in each chapter, including videos, motion graphics, audio, and text. A knowledge check is performed at the end of each chapter. Learners have unlimited attempts to pass the knowledge check and cannot proceed until a grade of 100% is achieved. Once the knowledge check is passed, the learners are presented with an animated video to acknowledge their avatar’s new rank (Figure 5).

As part of the implementation phase, we decided not to market or promote the intervention on the internet actively, limiting the number of participants and enabling us to pilot the intervention. The data collected from this first pilot population will be considered for the first iteration of the analysis phase, which will form the basis for answering the research questions described in this protocol.

### Measuring Change in Vaccine Hesitancy

Various scales have been developed to measure vaccine hesitancy among parents or health care workers [17]. The World Health Organization Strategic Advisory Group on Experts Working Group on Vaccine Hesitancy developed a 10-item Vaccine Hesitancy Scale that is widely used in different countries and settings [24]. The Vaccine Hesitancy Scale has been modified to the aVHS and has been adapted and validated in English and Chinese [17]. The aVHS is a 10-item scale with a 5-point Likert scale ranging from “Strongly disagree” to “Strongly agree.” The Likert scale items have scores ranging from 10 to 50, where 50 represents the highest degree of vaccine hesitancy, and 10 represents the lowest degree of vaccine hesitancy. Further, 7 items on the scale will be reverse coded, so that the highest scores reflect the highest degree of vaccine hesitancy [17]. A cutoff score of 24 will be used to dichotomize the outcomes into the “vaccine hesitant” and “not vaccine hesitant” categories. All scores and cutoffs will be adopted by following the methodology of the research team developing the scale [17].

### Data Collection

The study will use a quasi-experimental design (ie, a pre-post test using the aVHS) to understand if any changes have occurred in learners’ hesitancy to vaccinate. The aVHS is embedded in the intervention, and it will be completed by the learner before and after the intervention, with results being stored on a secure server. In addition, analytics data are collected automatically by MBRU Learn, which records how learners engage with the intervention, including the amount of time they spent using the intervention.

All data will be collected within MBRU Learn, which is securely hosted locally at the MBRU. Therefore, only the research team will have access to the data. The data collection instruments will include the aVHS, and analytics of interactions with the intervention and sociodemographic data, including participants’ age range, country of residence, gender, and vaccination status, will be collected. Participants will also be asked about how they heard about the course.

### Recruitment

MBRU Learn is a web-based space that hosts all of our publicly available web-based interventions, enabling participants to locate and enroll in interventions quickly and free of charge. We will not actively recruit participants to the intervention for the study described in this protocol. As such, participants will include those who find the course by coming to the MBRU Learn platform either through previous experience with the platform or by word of mouth. After the revised intervention is released, we will actively market the course to the public through social media and email marketing.

### Ethics Approval

The study was approved by the university’s institutional review board in October 2021 (submission number: MBRU IRB-2021-68).

### Data Analysis

#### Sample Size

An a priori sample size calculation was conducted by using G*power (Universität Düsseldorf). A total sample size of 343 has a power of 95% (1 − *β* error probability) and an α error probability of 5% for detecting an effect size of 0.2 (20%).

#### Data Analysis Plan

Our data analysis plan describes how we will answer the research questions related to how learners engage with the course and any vaccine hesitancy changes. Learner engagement will be quantitatively analyzed by using the analytics data collected in MBRU Learn. Our analysis will focus on learner engagement and retention analytics—the two most common measurements that are used to understand and improve web-based interventions [25]. Learner engagement will be determined by analyzing analytics such as the time spent on lessons and activities, engagement with digital resources, the number of attempts for quizzes and assessments, the number of times content was viewed, and the amount of time spent overall on the intervention. Retention will be determined by understanding the overall completion rates. Recommendations for revisions to the course will be based on the analysis results. Next, we will use these analysis results and existing literature to make determinations about how the intervention can be made more effective, after which we will enter another iteration in the DBR process to output an improved intervention that will be heavily marketed on social media and to our existing database of over 1 million learners.

Impacts on vaccine hesitancy will be analyzed based on the pre-post aVHS test scores. Statistical analysis software will be used to analyze the data set, and a complete case analysis approach will be adopted. Data will be tested for normality visually, by using histograms, and statistically, by using the Shapiro-Wilk test. The significance level cutoff will be set at *P*<.05, and exact *P* values will be reported. Continuous variables will be further categorized after data visualization to avoid having groups with sparse data. Means, medians, IQRs, and SDs will be used to describe continuous variables. For categorical or nonnormally distributed variables, a Wilcoxon signed rank test will be used. Frequency distributions, percentages, and chi-square tests will be used to describe binary and categorical variables and identify any significant differences.

The correlation between vaccine hesitancy scores and the vaccination statuses reported by participants will also be explored. aVHS scores will be summed (range: 10-50), and hesitancy will be determined according to the tool’s design (scores of 10-24 will be categorized as “not hesitant,” and scores of 25-50 will be categorized as “hesitant”). The primary outcome results will be presented as proportions of vaccine-hesitant individuals with the corresponding 95% CIs. The vaccine hesitancy statuses will be stratified by participants’ gender, age range, and country of residence. A pre-post comparison of the responses to the aVHS will be conducted by using a Wilcoxon signed rank test. ANOVA analyses and regression models will be constructed to evaluate the potential confounding effect that arises from variations in the sociodemographic factors of participants. The significance level cutoff will be set at *P*<.05, and exact *P* values will be reported.

## Results

The study described in this protocol is part of a larger research project titled “Addressing Vaccine Hesitancy through Targeted and Personalized Mobile Educational Interventions for Different Populations in the Eastern Mediterranean region.” The university’s institutional review board approved the study in October 2021 (submission number: MBRU IRB-2021-68). Data collection began in September 2021, and it is ongoing. This protocol describes the study, which will use data collected from September 2021 to January 2022, as outlined in our institutional review board approval. Once the analysis is complete and another round of DBR has been conducted, the improved intervention will be released, and promotion activities will commence to recruit participants. The study outcomes are expected to be published by August 2022. We anticipate the release of the revised intervention by May 2022. In addition, any work resulting from our study will be disseminated nationally and internationally through submission to academic journals and international conferences.

## Discussion

### Study Implications

Population health education plays a crucial role in the prevention and control of diseases. Methods of population health education are varied and can consist of simple awareness campaigns, pamphlets, advice from health professionals, or web-based education interventions [9]. The factors to consider when designing health education to address pandemics include addressing the fear and stigma that members of the public might feel [26], which can contribute to dangerous behaviors, such as denying infection and delaying health care uptake [9]. The World Health Organization developed guidelines to address the issue of social stigma, and one of the main components is “spreading the facts,” which explains that stigma is enhanced with insufficient knowledge about a disease’s mode of transmission, treatment, and prevention. This could be rectified via the collection and dissemination of sound and accurate information [27]. It was found that anxiety levels significantly decreased after adequate health education [9] and awareness improved. Hence, the learning design of our intervention includes digital literacy skills for identifying misinformation and wellness techniques that can help address issues of anxiety related to COVID-19 and vaccines. Our health education intervention aims at improving knowledge and perceptions and empowering individuals by increasing self-efficacy among the general population. The web-based course is publicly available, and it will be continuously improved and updated to address areas of concern regarding COVID-19. It serves as a reliable resource for individuals who have questions and concerns that need to be addressed in a creative and interactive way.

As discussed previously, for web-based interventions to succeed in changing behaviors, considerations of learning design and motivation strategies must be undertaken. However, due to the limitations within our platform and a lack of available resources, we cannot use typical learning and motivation strategies, such as establishing a social presence on discussion forums or through group work, to motivate learners to finish our courses [28,29]. Hence, in our learning design, we use strategies such as reflection, which helps connect learners to their prior experiences and establish relevance; use the provision of immediate feedback on knowledge checks to support competence; create an engaging experience through variations in learning activities and digital content; and use gamification principles, such as a progression strategy, whereby the intervention is told through a story in which the learner is the hero [14,30,31].

In their systematic review of papers on using gamification for MOOCs, de Freitas and da Silva [25] found that studies that used gamification principles reported greater participation from learners, as reflected by the time spent on MOOC platforms, the number of learners completing end-of-course evaluations, and the number of tasks and lessons completed [25]. Gamification in education involves applying game design elements to improve learner engagement and motivation [32]. Gamification is commonly linked with self-determination theory, which looks at the concept of motivation, focusing on individuals' intrinsic (internal) and extrinsic (external) motivation [33]. The characteristics of intrinsic motivation include curiosity and interest in learning new things [34]. It has been recommended that interventions that use gamification strategies be carefully designed to balance considerations for intrinsic and extrinsic motivation [34,35]. Given that our educational intervention is entirely voluntary and is not part of a formal curriculum, it can be safely assumed that our learners are intrinsically motivated. As such, our educational intervention uses extrinsic motivation techniques to stimulate our learners toward completing the entirety of the intervention and sharing their accomplishments via social media.

### Limitations

The limitations in the study are related to the current intervention and the dissemination of the intervention thus far. The current intervention is only available in English, limiting our participant population. The intervention also only exists on the internet, limiting our participant population to those with digital literacy and internet access. Additionally, no personal or sociodemographic data will be collected from the participants until the end of the course, limiting these data to those from participants who achieve 100% completion. We have also chosen not to publicly disseminate the course, limiting the participants to those who are likely already followers of other community health courses in MBRU Learn or those who advocate for our courses already. This can skew the completion statistics, as these participants may be highly motivated to participate in the intervention. However, analytic observations of highly motivated participants are valuable measures, as they may help us identify larger errors more easily in our design. Despite this population limitation, we believe that its impact on our understanding of learner engagement will be limited and that the analysis of vaccine hesitancy status via the aVHS will not experience the same bias. Current and subsequent iterations of the intervention will only be available on MBRU Learn, which might only reach a limited demographic. However, our future direction is to use Google ads to promote the intervention to a larger population. The absence of an adequate control group is also another limitation in the study’s ability to assess the impact of the intervention on vaccine hesitancy, which will otherwise be measured via a pre-post scale. Adopting a pre-post survey (quasi-experimental design) is very common, especially in studies on educational interventions. The assumption is that the impact of the knowledge gained will be identified based on the changes in the test scores. One of the known limitations of this design is the use of a pretest, which potentially informs participants about the matter of interest and allows them to score better on a posttest rather than acquire adequate general knowledge on the subject of interest [36]. This has been controlled for in our study by blinding the participants to the scores of the scale and the interpretation of those scores. Also, participants act as their own controls in the study design. This controls for other potential confounding factors.

### Conclusions

Our study will explore the use of a web-based educational intervention that is designed to address vaccine misinformation and observe any changes in learners’ hesitancy to vaccinate. The results of the study will contribute to using evidence-based practices to better understand how to develop public health interventions that contribute to positive health behavior changes, such as the willingness to vaccinate and the identification of public health misinformation. Any work resulting from this project will be disseminated nationally and internationally through submission to academic journals and international conferences.

## Abbreviations

aVHS: Adult Vaccine Hesitancy Scale
DBR: design-based research
ieHPE: Institute for Excellence in Health Professions Education
MBRU: Mohammed Bin Rashid University of Medicine and Health Sciences
MERS: Middle East respiratory syndrome
MOOCs: massive open online courses
SARS: severe acute respiratory syndrome
SME: subject matter experts
